# A computational model to predict bone metastasis in breast cancer by integrating the dysregulated pathways

**DOI:** 10.1186/1471-2407-14-618

**Published:** 2014-08-27

**Authors:** Xionghui Zhou, Juan Liu

**Affiliations:** School of Computer, Wuhan University, Wuhan, P.R. China

**Keywords:** Bone metastasis, Breast cancer, Dysregulated pathways, Prediction model, Immune system

## Abstract

**Background:**

Although there are a lot of researches focusing on cancer prognosis or prediction of cancer metastases, it is still a big challenge to predict the risks of cancer metastasizing to a specific organ such as bone. In fact, little work has been published for such a purpose nowadays.

**Methods:**

In this work, we propose a Dysregulated Pathway Based prediction Model (DPBM) built on a merged data set with 855 samples. First, we use bootstrapping strategy to select bone metastasis related genes. Based on the selected genes, we then detect out the dysregulated pathways involved in the process of bone metastasis via enrichment analysis. And then we use the discriminative genes in each dysregulated pathway, called as dysregulated genes, to construct a sub-model to forecast the risk of bone metastasis. Finally we combine all sub-models as an ensemble model (DPBM) to predict the risk of bone metastasis.

**Results:**

We have validated DPBM on the training, test and independent sets separately, and the results show that DPBM can significantly distinguish the bone metastases risks of patients (with p-values of 3.82E-10, 0.00007 and 0.0003 on three sets respectively). Moreover, the dysregulated genes are generally with higher topological coefficients (degree and betweenness centrality) in the PPI network, which means that they may play critical roles in the biological functions. Further functional analysis of these genes demonstrates that the immune system seems to play an important role in bone-specific metastasis of breast cancer.

**Conclusions:**

Each of the dysregulated pathways that are enriched with bone metastasis related genes may uncover one critical aspect of influencing the bone metastasis of breast cancer, thus the ensemble strategy can help to describe the comprehensive view of bone metastasis mechanism. Therefore, the constructed DPBM is robust and able to significantly distinguish the bone metastases risks of patients in both test set and independent set. Moreover, the dysregulated genes in the dysregulated pathways tend to play critical roles in the biological process of bone metastasis of breast cancer.

**Electronic supplementary material:**

The online version of this article (doi:10.1186/1471-2407-14-618) contains supplementary material, which is available to authorized users.

## Background

Metastasis is the main cause of death in breast cancer [1, 2], and bone is the organ suffering from metastasis most frequently [3]. Breast cancer patients with bone metastases may suffer marked decreased mobility, pathologic fractures, neurological damage and other symptoms, and the patients with high risks of bone metastases should take agents tailored treatments [4, 5]. Thus for cancer therapy, it is essential to identify the prognostic factors which can help to identify the patients with high risks of bone metastasis [4–6].

Because the ability of tumour cells metastasizing to a specific organ is an inherent genetic property [7, 8], it is possible to predict bone metastasis of breast cancer by using gene expression profiles [8]. However, up to now only several researches have attempted to identify bone metastasis related genes from gene expression data [3, 9–11], and only one in which [3] has made use of the identified genes as signature to construct classification model for predicting bone metastasis risk of breast cancer. What is more, the published work just considered very limited number of samples when selecting gene signatures and did not perform strict independent tests on any larger data set. As breast cancer is a heterogeneous disease, the characters associated with metastases may vary widely across different patients [1]. Insufficient patient samples would not cover all aspects of the metastases, thus gene signatures selected from small number of samples may not be credible enough. In fact, it has been found out that the gene signatures identified using one data set may perform badly on another data set [12–14].

**Figure 1:**
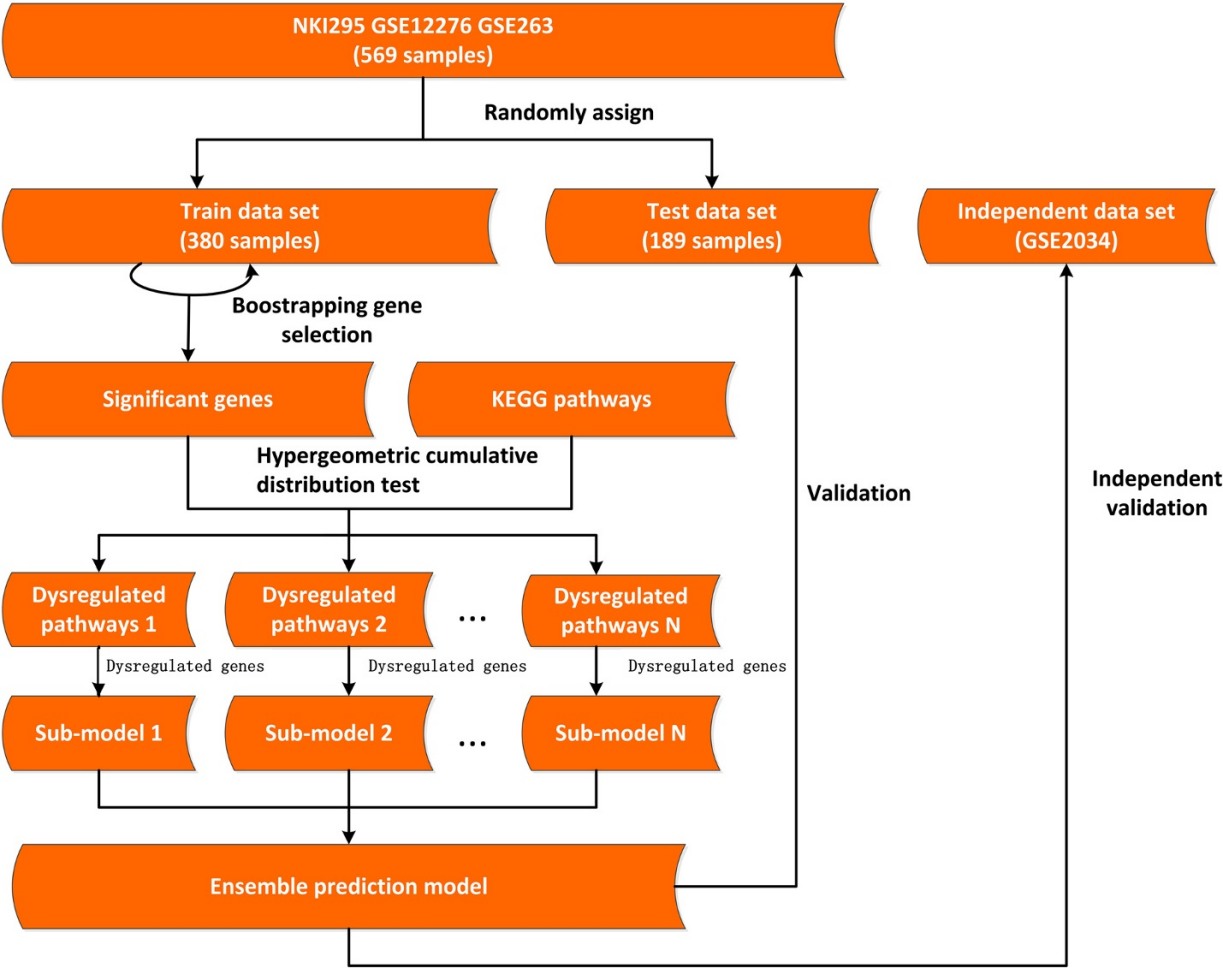
**The framework of DPBM prediction model.**

In recent years, several methods have been used to derive gene sets that are related to specific biological functions, such as protein-protein interaction network [15], pathway [16], GO Term [17], and so on. For example, the gene set statistics method [17] infers the activity of one gene set by counting all expression levels of genes in the set, and then uses the activity to build the classifier to predict the metastasis risk of breast cancer. Extracting gene sets rather than selecting single genes can provide more stable signatures, thus can construct classifiers with higher performances [18]. However, most of the existing methods consider all genes in the same set equally without noticing that some genes are less important than others. In fact in a pathway or other kind of gene set, only a part of genes would be dysregulated during the metastasis process of cancer. Although Lee *et al*. just considered a subset of the genes to infer the activity of each pathway, and used all activities to construct a model to classify cancer patients [18], there are still two drawbacks. Firstly, this method uses the inferred activities instead of the gene expression levels to construct the classifier, resulting in the loss of some important information for classification. Secondly, some pathways not involved in the disease process may be considered improperly, leading that some noises could be imported into the prediction model.In this work, we present a new prediction model, Dysregulated Pathway Based prediction Model (DPBM), to predict the risk of bone metastasis of breast cancer (Figure 1). To get enough samples, we integrate four breast cancer sets together to obtain 855 breast cancer samples, from which we select genes that are significantly correlated with bone metastasis of breast cancer by using bootstrapping strategy. The selected genes are also called as candidate genes. After that, we identify KEGG pathways that are enriched by the candidate genes as abnormal pathways in the bone metastasis process. We call these pathways as dysregulated pathways and the candidate genes involved in the dysregulated pathways as dysregulated genes. Since different pathways are involved in different aspects of the bone metastasis process, the genes related to them can correspondently be divided into different functional groups. Therefore, we can use the dysregulated genes in each pathway to construct one sub-model, and then integrate all sub-models into an ensemble model (DPBM) to predict the bone metastases risks of breast cancer patients by majority voting strategy. We evaluate DPBM both on test set and independent set in terms of prediction accuracy and robustness. We also investigate the topological characteristics of the dysregulated genes in protein-protein interaction network and their functional annotations, trying to uncover the biological mechanisms that play important roles in bone metastasis of breast cancer.

## Methods

### Data sets and pre-processing

We have downloaded gene expression profiles of breast cancer patients along with the clinical information from UNC microarray database [8]. The downloaded data consists of four microarray data sets: GSE2034 [19], GSE2603 [20], GSE12276 [21] and NKI295 [22], and has been processed and normalized by the original paper [8]. Details of these data sets are shown in Table 1. In our work, GSE2034 was used as an independent test set. As for the other three data sets, we randomly selected 2/3 samples as the training set and the remainder samples as the test set. As a result, we got a training set consisting of 380 samples (113 are bone metastases and 267 are free of bone metastases) and a test set containing 189 samples (56 are bone metastases and 133 are free of bone metastases). In these data sets, if the first metastasis organ of a patient is bone, then the status is set as bone metastasis, otherwise it is set as free of bone metastasis (including cases of non-bone metastases and non metastases).

**Table 1:** **Breast cancer data sets**

Data set	Bone metastasis samples	Metastasis samples	Samples
GSE2034	69	95	286
GSE2603	14	24	82
GSE12276	102	173	192
NKI295	53	84	295

GSE2034 was used as an independent set. The other three data sets were combined into one merged set, from which we randomly selected 2/3 samples into the training set and the other 1/3 samples into the test set.

We have also downloaded the human protein-protein interactions from the HIPPI (Human Integrated Protein-Protein Interaction rEference) [23], and the pathways from the Molecular Signatures Database (MSigDB) [24].

### Selecting candidate genes by bootstrapping

As is known to all, t-test is a popular method used to select discriminative genes, thus it could be used in our work. However, t-test method requires that every sample must be attached with a class label. While in our work, for the reason that the clinical information of some patients is censored, not every sample can be assigned as either low-risk or high-risk of bone metastasis according to the widely used criterion that patients who are bone-metastasized within a threshold of years belong to high-risk group, and patients who are free of bone metastases and survive longer than the threshold belong to low-risk group, which results that some valuable samples not satisfying the criterion have to be removed from the training set if t-test method is used. Different with t-test method, however, the Cox proportional hazards regression can involve all samples into the calculation, thus it is more proper for our work to select the bone metastasis related genes.

In this work, we used a simple bootstrapping strategy to select candidate genes of which expression levels were significantly correlated with the bone metastasis risk. Concretely, we first randomly selected 3/4 of all the 380 samples from the training set; and then for each gene, we applied Cox proportional hazards regression to calculate the coefficient between the gene expression level and the bone metastasis risk across the chosen samples. The above procedure was repeated 400 times, and the genes with Cox p-values less than 0.05 in more than 80% of all runs were regarded as the candidate genes. For every selected gene, its averaged Cox coefficient and Cox p-value over all the 400 runs were set to be its final corresponding values for further calculations.

### Identifying the dysregulated pathways

The candidate genes are those significantly correlated with bone metastasis risk. If the candidates are enriched in a pathway (that is, the overlap of the candidate genes and the genes in the pathway is significant), then we call this pathway as a dysregulated pathway. In this work, we applied the widely used hyper geometric cumulative distribution function to test the significance of the overlap:


Where *x* stands for the size of intersection set; *K* represents the number of the candidate genes; *N* stands for the number of the genes in the pathway; and *M* represents the number of all genes in our calculation (the universal gene set). For a pathway, if the *p*-*value* is less than 0.05, then it is considered as the dysregulated one; and the genes belonging to the intersection set are called as dysregulated genes.

### Constructing the DPBM

With the hypothesis that one dysregulated pathway may describe only one aspect of the bone metastasis mechanism, while all dysregulated pathways can provide a comprehensive view of the bone metastasis, we adopted the ensemble strategy [14] to construct DPBM to predict the bone metastases risks of breast cancer patients. We chose the dysregulated genes in each dysregulated pathway as features to construct a sub-model to distinguish the bone metastases risks of the patients, and all the sub-models were integrated as DPBM by majority voting strategy.

To construct each sub-model, we used a simple strategy, similar to the Gene expression Grade Index (GGI) [25], to calculate the bone metastasis risk for every patient, shown as the following equation:


Where *x*_*i*_ (*x*_*j*_) represents the expression level of the dysregulated gene *i* (*j*) which has a positive (negative) Cox coefficient with metastasis risk. The higher the *RiskScore* is, the greater the risk of bone metastasis. We applied 10-fold cross validation test to set the proper threshold value of *RiskScore*. In each run, the *n*-*th* smallest *riskScore* value (*n* is the number of training patients free of bone metastases) in the training samples was set as the cut-off to determine the class labels of the test samples, based on which, the performance (log rank test) can be obtained. The final threshold value was set as the one with the best performance in ten runs. Any patient with *RiskScore* value greater than this threshold is considered as high-risk of bone metastasis by this sub-model, otherwise it is considered as low-risk of bone metastasis.

For a patient, if more than half sub-models vote for “high-risk of bone metastasis”, it will be finally predicted as “high-risk of bone metastasis” by DPBM, and vice versa. In order to assess the performance of DPBM, we used the log rank test to evaluate the significance of the risk differences between the patients in two groups. Kaplan Meier curves and the log rank test were performed using a tool (http://www.mathworks.com/matlabcentral/fileexchange/22317-logrank).

### Topologically investigating dysregulated genes in PPI network

Protein-protein interaction network has been successfully applied to select signature genes [26]. For example, Hase *et al*. illustrated that the signature genes tended to have bigger degrees in the network [27]; and Yao *et al*. reported that the signature genes were usually with higher betweenness centralities in the network [28]. Thus we investigated two network topological coefficients (Degree and Betweenness Centrality) of the selected dysregulated genes by comparing with candidate genes (dysregulated genes excluded) and all genes in the PPI network (dysregulated genes excluded). The differences of the topological coefficients between the dysregulated genes and other two kinds of genes were tested by the Mann–Whitney-Wilcoxon non-parametric test for two unpaired groups. And the topology analysis of PPI network was performed by the Network Analyzer plug-in for Cytoscape [29].

### Investigating dysregulated genes by functional analysis

DAVID [30] was applied to extract the GO Terms (Biological Processes) which were significantly enriched by the dysregulated genes and the ones with p-values less than 0.05 were set as enriched GO Terms. All enriched GO Terms were clustered into several functional groups by the functional annotation clustering method with the default threshold of enrichment score [30].

## Results

### Dysregulated pathways and genes

By bootstrapping method, we selected out 267 candidate genes (Additional file 1: Table S1), from which we got 35 dysregulated genes involved in eight dysregulated pathways (Table 2). In order to validate our strategy, we also used t-test to select the discriminative genes between the patients of the high-risk group and the low-risk group (see Additional file 1: Supplementary Methods), based on which, the dysregulated genes as well as dysregulated pathways can be gotten by using the similar strategy to ours. As a result, most of the identified dysregulated pathways and genes based on the candidates selected by bootstrapping method are significantly coincident with those selected by t-test method (Additional file 1: Figure S1). Moreover, most of the dysregulated pathways and genes are shown to be related to bone metastasis in literature.

**Table 2:** **The dysregulated pathways**

KEGG pathway	Enrichment p-value	Gene ID	Gene symbol	Cox coefficient	Cox p-value	Stability
Cytokine Cytokine Receptor Interaction	0.029	355	FAS	−0.42	0.0048	0.9925
		1235	CCR6	−0.22	0.023	0.905
		1439	CSF2RB	−0.32	0.0046	0.9875
		2322	FLT3	−0.28	0.015	0.93
		3561	IL2RG	−0.20	0.031	0.8125
		3570	IL6R	−0.37	0.027	0.85
		3575	IL7R	−0.23	0.0044	0.995
		4982	TNFRSF11B	−0.22	0.019	0.9125
		6363	CCL19	−0.11	0.033	0.8025
		6375	XCL1	−0.21	0.013	0.9575
		7042	TGFB2	−0.24	0.016	0.9325
		7422	VEGFA	0.15	0.031	0.83
Chemokine Signaling Pathway	0.041	112	ADCY6	0.34	0.032	0.8225
		1235	CCR6	−0.22	0.023	0.905
		3702	ITK	−0.18	0.023	0.87
		3717	JAK2	−0.48	0.0025	1
		5579	PRKCB1	−0.39	0.021	0.8975
		5613	PRKX	−0.26	0.031	0.815
		5829	PXN	0.34	0.021	0.8725
		6363	CCL19	−0.11	0.033	0.8025
		6375	XCL1	−0.21	0.013	0.9575
Cell Cycle	0.012	894	CCND2	−0.27	0.013	0.96
		1021	CDK6	−0.48	0.010	0.955
		1869	E2F1	0.32	0.0015	1
		1870	E2F2	0.33	0.031	0.8375
		7042	TGFB2	−0.24	0.016	0.9325
		8243	SMC1A	0.75	0.0085	0.98
		9700	ESPL1	0.25	0.010	0.97
		10744	PTTG2	0.46	0.011	0.975
Natural Killer Cell Mediated Cytotoxicity	0.048	355	FAS	−0.42	0.0048	0.9925
		3002	GZMB	−0.23	0.0067	0.985
		3383	ICAM1	−0.32	0.022	0.8975
		3821	KLRC1	−0.43	0.0073	0.97
		3932	LCK	−0.24	0.025	0.875
		5579	PRKCB1	−0.39	0.021	0.8975
		22914	KLRK1	−0.34	0.015	0.9325
T Cell Receptor Signaling Pathway	0.046	917	CD3G	−0.43	0.00097	0.9975
		3702	ITK	−0.18	0.023	0.87
		3932	LCK	−0.24	0.025	0.875
		5788	PTPRC	−0.21	0.024	0.8675
		10892	MALT1	−0.43	0.015	0.905
		29851	ICOS	−0.41	0.018	0.915
Pancreatic Cancer	0.027	1021	CDK6	−0.48	0.010	0.955
		1869	E2F1	0.32	0.0015	1
		1870	E2F2	0.33	0.031	0.8375
		7042	TGFB2	−0.24	0.016	0.9325
		7422	VEGFA	0.15	0.031	0.83
Non Small Cell Lung Cancer	0.0095	1021	CDK6	−0.48	0.010	0.955
		1869	E2F1	0.32	0.0015	1
		1870	E2F2	0.33	0.031	0.8375
		5579	PRKCB1	−0.39	0.021	0.8975
		6256	RXRA	0.45	0.012	0.9525
Primary Immunodeficiency	0.0014	3561	IL2RG	−0.20	0.031	0.8125
		3575	IL7R	−0.23	0.0044	0.995
		3932	LCK	−0.24	0.025	0.875
		5788	PTPRC	−0.21	0.024	0.8675
		29851	ICOS	−0.41	0.018	0.915

The first column contains the names of the pathways; the second column contains the enrichment p-value of the candidate genes to the pathways; the third column (Gene ID) and the forth column (Gene Symbol) contains all candidate genes in the pathways; the fifth column contains the average Cox coefficients of the genes in the 400 runs; the fifth column contains the average p-values of the genes in the 400 runs and the last column contains the stability of the genes in the 400 runs (the ratios of the genes are significant across all the 400 runs). In the table, there are 35 unique genes (some genes may be present at more than one pathways).

Some cytokines have been reported to be related to breast invasion and metastasis site [31], while cytokine receptor interaction pathway has been found significant in our work. What is more, the dysregulated genes IL2RG, IL6R, IL7R and TGFB2 have been reported to be associated with metastasis site or prognosis [31], and CCR6 is associated with both live metastasis in breast cancer [32] and bone metastasis in human neuroblastoma [33].

Chemokines and their receptors have been shown to play critical roles in determining the metastatic destination of tumour cells [34]. In our work, the chemokine signalling pathway is also enriched with the candidate genes. In the meanwhile, among the nine dysregulated genes, Jak2 has been reported to be mediated by IL6 to involve in bone metastasis [35]; CCR6 is associated with bone metastasis [33]; PPKX regulates endothelial cell migration and vascular-like structure formation [36]; XCL1 and CCL19 are associated with organ specific metastasis [34, 37].

Cell cycle pathway plays an important role in tumorigenesis and cancer prognosis [38], and it has also been found to be dysregulated in our work. Among its dysregulated genes, CCND2 is differentially expressed between breast cancer patients with bone metastases and other patients [11]; E2F1 can regulate DZ13 to induce a cytotoxic stress response in tumour cells metastasizing to bone [39]; TGFB2 is related to the bone metastases development [40].

It is interesting that non-small cell lung cancer and pancreatic cancer pathways have also be found dysregulated in bone metastasis. In fact, lung is the organ with the second frequent metastasis for breast cancer [8], and it has been reported that some breast cancer would metastasize to pancreatic [41]. This phenomenon suggests that either lung cancer or pancreatic cancer might share some common mechanisms with bone metastasis of breast cancer, for the dysregulated genes E2F1 [39] and TGFB2 [40] in pancreatic cancer pathway have been shown to be also involved in bone metastasis process; while E2F2 gene, the family member of E2F1, has been found to be the dysregulated gene in the non-small cell lung pathway.

We have also found that three immune related pathways have been dysregulated in bone metastasis of breast cancer: natural killer cell mediated cytotoxicity pathway, T cell receptor signalling pathway and primary immunodeficiency pathway. In fact, some immune related genes are essential in bone metastasis of breast cancer [42–44], and their family members, such as FAS, IL2RG and IL7R, have shown dysregulated in our work and have been reported to be either metastasis related or bone metastasis related [31, 35, 45].

Now that references [3, 9–11] have published bone metastasis related genes, we merged all the reported genes and investigated the overlap with our dysregulated genes. It is surprising that there are only four common genes (Additional file 1: Figure S2) between two sets of genes. We thus investigated the functions of published genes and found that they are most enriched in ‘metabolic process’ (data not shown), while our dysregulated genes are mainly related to immune system. By literature investigation, we further found that the immune cells can play essential roles in bone metastasis or metastasis of cancer [42, 44], which illustrates that our dysregulated genes are related to some new biological mechanism of bone metastasis, compared to the reported genes.

### Distinguishing bone metastasis risk by DPBM

**Figure 2:**
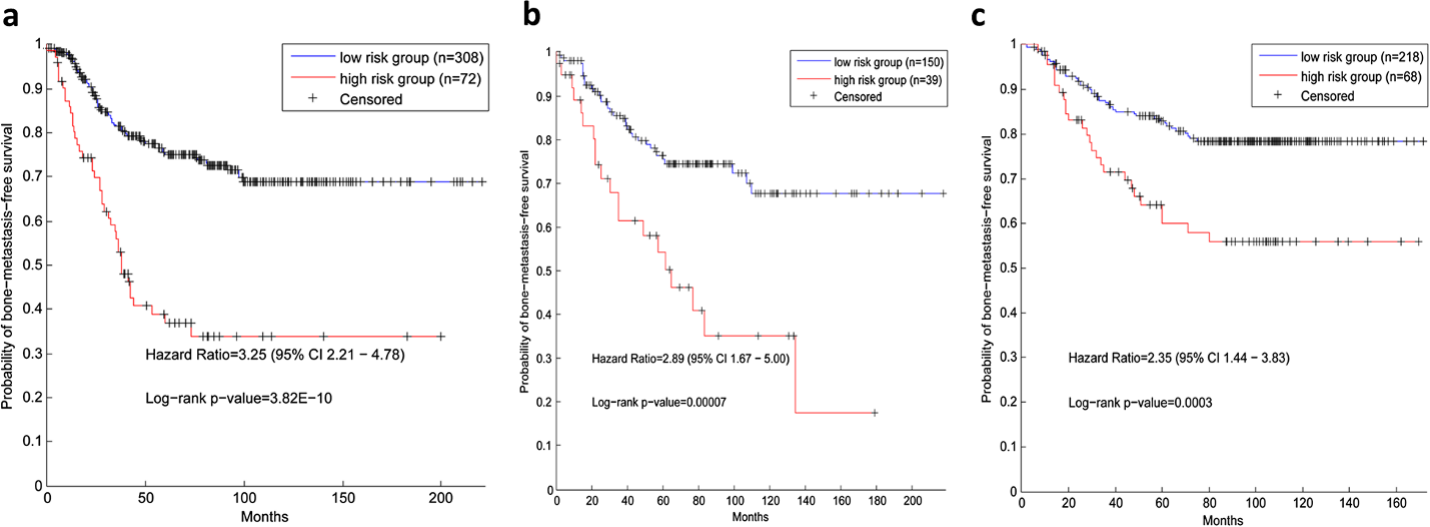
**Kaplan**-**Merier curves of the risk groups for breast cancer patients with bone metastasis**-**free survival. (a)** Result in the training set. **(b)** Result in the test set. **(c)** Result in the independent set.

From the training set we have extracted eight dysregulated pathways for bone metastasis in breast cancer, based on which, eight sub-models were constructed and then integrated into DPBM for predicting the bone metastases risks of patients. Therefore, we decided to evaluate DPBM on the training set, test set and independent set respectively.Just as expected, DPBM performed well in the training set. Among all the 380 patients, 308 have been classified as low-risk of bone metastases, and 72 as high-risk of bone metastases. The hazard ratio of the two groups was 3.25 (95% CI 2.21 – 4.78), with p-value of 3.82E-10 (Figure 2a).Then we validated DPBM on the test set and found it also performed very well. Among the 189 patients, 150 samples were predicted as low-risk and the others as high-risk. Survival analysis showed that the hazard ratio was 2.89 (95% CI 1.67 – 5.00), with p-value of 0.00007 (Figure 2b).It is notable that both the training and test sets belong to the same integrated data set, the test set is hardly independent with the training set even though it has not taken part in the construction of DPBM. Therefore, it would be bias to evaluate DPBM just with the test set or even with the training set. Herein, we also used a completely independent set, GSE2034, to evaluate DPBM. The result shows that DPBM consistently performed well in the independent set. Among the 286 samples, 218 patients were predicted as low-risk group and the other 68 ones were assigned into the high-risk group. The hazard ratio between the two groups was 2.35 (95% CI 1.44 – 3.83), and the p-value of log rank test was 0.0003 (Figure 2c).

We noticed that different types of samples in any of the training, test and independent sets are imbalanced, which would lead to the overestimation problem. In order to address this issue, we also used random sampling methodology to choose the same number of cases from high-risk and low-risk groups and re-evaluated the DPBM on each of three data sets. We repeated the above process 1000 times, and the means of hazard ratios for training test and independent sets were 3.31 (p-value of 2.49E-04), 3.15 (p-value of 0.0082) and 2.48 (p-value of 0.015) respectively (Additional file 1: Table S2). The results further unveil the robustness of our model. In the meanwhile, the stable performance of the DPBM also indicates the reliability of the dysregulated genes identified by our method.

### Topological analysis of dysregulated genes in PPI network

**Figure 3:**
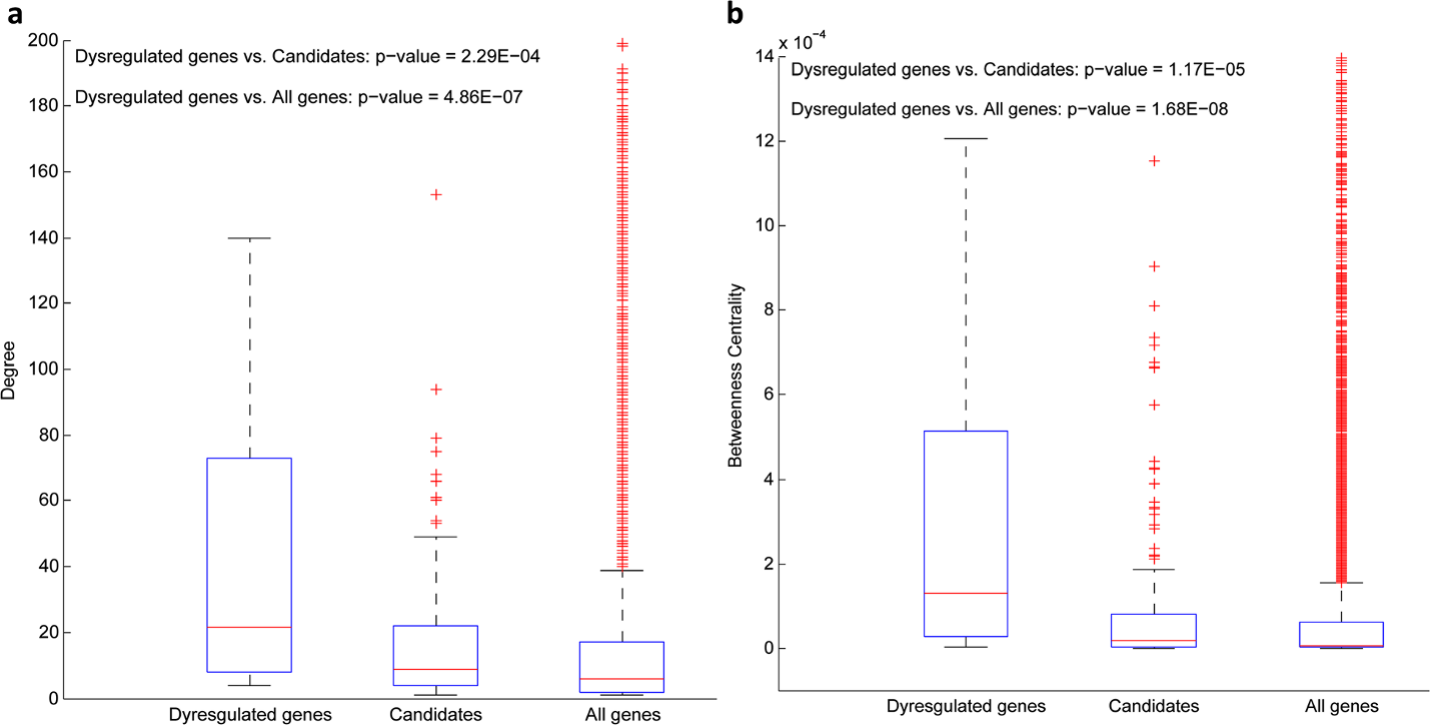
**Comparison of the topological parameters in the PPI network among the three groups (Dysregulated genes,** **Candidate genes (except for the dysregulated genes) and All genes (except for the dysregulated genes) in the PPI network). (a)** Comparison of the degrees. **(b)** Comparison of the betweenness centralities.

The degrees and betweenness centralities of three groups of genes (35 dysregulated genes, 232 candidate genes (the dysregulated genes excluded), all genes (the dysregulated genes excluded) in PPI network) are shown in Figure 3(a) and Figure 3(b) respectively, where three gene groups are correspondingly denoted as ‘Dysregulated genes’ , ‘Candidate genes’ and ‘All genes’.

From Figure 3(a), it is clear that the dysregulated genes tend to have bigger degrees than the other two groups of genes, and the p-values of dysregulated *vs* candidate genes, dysregulated *vs* all genes are 2.29E-04 and 4.86E-07 respectively. Moreover, Figure 3(b) demonstrates that the betweenness centralities of the dysregulated genes are usually bigger than the other two groups of genes (with p-value = 1.17E-05 and p-value = 1.68E-08 separately).

From above results we can see that the dysregulated genes take up more important positions in the PPI network than the other genes, and tend to be essential genes for the bone metastasis.

### Difference between bone and non-bone metastasis

We noticed that there are also some samples metastasized to other organs instead of bone in the data sets. By using the same strategy as we have done for bone metastasis, we have found nine dysregulated pathways and a total of 67 dysregulated genes related to non-bone metastases (metastases to other organs except for bone) (Additional file 1: Table S3). Therefore, we investigated the different functional groups to which these two kinds of genes belong, with the purpose of uncovering the biological mechanism of bone specific metastasis. By function annotating and clustering, the 35 dysregulated genes of bone metastasis were found to belong to 16 functional groups (Additional file 2: Table S4), and the 67 dysregulated genes of non-bone metastases were found to belong to 15 functional clusters (Additional file 3: Table S5).

By comparison, we found that these two kinds of genes shared a lot of common functional clusters. For example, cell differentiation related cluster, cell cycle related cluster, cell migration cluster, apoptosis related cluster, hormone stimulus related cluster, phosphate metabolic process and phosphorylation related cluster. As is known to all, cell differentiation, cell cycle, cell migration, and cell apoptosis are all famous caner hallmark related GO Terms that are related to cancer and cancer prognosis [46–48], while hormones are related to the risk of breast cancer and hormones-replacement therapy is a common therapy for breast cancer patients [49]. In addition, phosphorylation of some proteins have been reported to be related to breast cancer [50] and cancer prognosis [51].

The main difference between these two kinds of dysregulated genes was that dysregulated genes of bone metastasis are also enriched in biological processes associated with immune system, whereas dysregulated genes of non-bone metastases were not. The difference suggests that the immune system may be essential in the bone specific metastasis of breast cancer.

### Comparing DPBM with other classification methods

In DPBM, we simply used a cut-off of the *RiskScore* in each dysregulated pathways to make a prediction, instead of training a complex classifier such as SVM (Support Vector Machine). In order to evaluate this option, we herein adopted two strategies to construct SVM classifers and investigated their performances. By one strategy, we used the *RiskScore* values of the eight dysregulated pathways as eight features to construct a SVM classifier. By the other strategy, we used all the 35 dysregulated genes as features to construct another SVM classifier to predict the bone metastasis risk. To construct both SVM classifiers, the patients in the training set were labelled as high-risk or low-risk as described in Additional file 1: Supplementary Methods. The performances of these two kinds of SVM classifiers are listed in Table 3. The comparing results indicate the superiority of DPBM even through it adopts a simple classification strategy.

**Table 3:** **Comparing DPBM with other methods**

	Training data set		Test data set		Independent data set	
	AUC	Accuracy	AUC	Accuracy	AUC	Accuracy
DPBM	0.76	0.64	0.60	0.60	0.61	0.66
SVM (*RiskScore*)	0.72	0.71	0.58	0.59	0.60	0.60
SVM (dysregulated genes)	0.75	0.75	0.55	0.54	0.60	0.59
SCC	0.78	0.65	0.57	0.55	0.57	0.44

As far as we know, there is only one published work to construct a model for predicting bone metastases risks of cancer patients [3], by using SCC (shrunken centroids classifier) [52] method. Therefore, we also compared DPBM with SCC. Since the data set used in the original work is too small, we constructed SCC and evaluated its performances on our data sets (the training samples were labelled as high-risk or low-risk as described in Additional file 1: Supplementary Methods, and 35 dysregulated genes were used as features). The results are also listed in Table 3, from which we can see that our DPBM performs better than SCC that has been used in previous work [3].

## Discussion and conclusions

Predicting the bone metastases risks for breast cancer patients is essential in cancer therapy, which is an urgent challenge now [5]. In this work, we have proposed a Dysregulated Pathway Based prediction Model (DPBM) to address this problem. We first selected the candidate genes (correlated with the bone metastasis) by bootstrapping strategy. Then we identified the dysregulated pathways enriched by the candidate genes. After that, we used the dysregulated genes in each dysregulated pathway to construct a sub-model to predict the bone metastasis risk separately. Finally, we combined all sub-models together by using majority voting strategy as an ensemble model, DPBM, to predict the risk of bone metastasis. Validation results on test set and independent set have shown the great prediction power of DPBM.

By literature investigation, most of the dysregulated pathways and dysregulated genes are related to bone metastasis. In addition, the dysregulated genes tend to have higher degrees and betweenness centralities in PPI network, suggesting that they play critical roles in the biological functions. By comparing the functional groups to which the dysregulated genes of bone and non-bone metastases belong, we found that the immune system may be essential in the bone specific metastasis of breast cancer.

All the results illustrate that the dysregulated genes may be good biomarker candidates. The facts that DPBM consistently performs well in both test set and independent set may be due to the following merits: (1) we used the pathways to filter the candidate genes, which can help to remove those genes less essential to the bone metastasis; (2) instead of selecting pathways or other functional gene sets via the activity differences between different phenotypes, we selected the dysregulated pathways enriched by the discriminative genes, which can help to preserve the useful information for classification and reduce noises; (3) we constructed one sub-model based on each dysregulated pathway, and then combined all sub-models by majority voting strategy. The ensemble classifier usually performs better than simple classifiers [53].

In this work, although we have collected 855 samples, the samples with the metastases to other specific organs are still insufficient, that is why we merged all samples with metastatic tumour of the other organs as one group (non-bone metastases group). This is reasonable for us to understand the difference between the bone metastasis and other organ metastases. Of course, if the samples with other organ metastases are sufficient, the differences among different metastases organs may also be well studied.

## Electronic supplementary material

Additional file 1: **This file contains two supplementary methods, three supplementary tables (Table S1 – Table S3) and two supplementary figures (Figure S1 – Figure S2).** (DOCX 474 KB)

Additional file 2: Table S4: (Functional clusters of dysregulated genes in the metastasis process to bone). This file describes the functional clusters of dysregulated genes involved in the bone metastasis process. (XLSX 46 KB)

Additional file 3: Table S5: (Functional clusters of dysregulated genes in the metastasis process to non-bone). This file describes the functional clusters of dysregulated genes involeved in the metastases processes to other organs. (XLSX 46 KB)
