# Supplementary material for: A computational model to predict bone metastasis in breast cancer by integrating the dysregulated pathways
Source: BMC Cancer. 2014 Aug 27;14:618. doi: 10.1186/1471-2407-14-618 (PMC4161863; doi:10.1186/1471-2407-14-618)
Supplement: Supplementary file 1 — Additional file 1: This file contains two supplementary methods, three supplementary tables (Table S1 – Table S3) and two supplementary figures (Figure S1 – Figure S2). (DOCX 474 KB) [file 12885_2014_4814_MOESM1_ESM.docx]

## Supplementary Methods

### Dividing patients into two groups of bone metastasis risks

We divided all the patients into high-risk or low-risk of bone metastasis group based on their bone-metastasis-free survival time as well as the bone-metastasis status. If a patient is bone metastasized within 8 years, then it belongs to high-risk group, and if the bone-metastasis-free survival time is more than 8 years, then the patient belongs to low-risk group. As a result, among all the 855 patients in the training set, 231 patients were set as high-risk groups and 245 patients were set as low-risk group, the other censored samples were discarded.

### Selecting dysregulated genes by t-test method

Based on method described above, we obtained 231 patients labeled with high-risk and 245 patients labeled with low-risk. We used t-test method to select out 1467 discriminative genes as candidates from the samples, by setting the threshold of p-value as 0.05. Based on the candidates, we applied enrichment analysis to identify the dysregulated pathways as well as dysregulated genes with the similar strategy of our DPBM method in the main text.

## Supplementary tables

**Table S1. All candidate genes which are directly correlated with the risk of bone metastasis**

| Gene ID | Cox coefficient | Cox p-value | Stability |
| --- | --- | --- | --- |
| 10058 | 0.379231447 | 0.0003166 | 1 |
| 10537 | -0.156002997 | 0.0005795 | 1 |
| 1869 | 0.316874492 | 0.0014567 | 1 |
| 23061 | 0.454439651 | 0.0037145 | 1 |
| 330 | -0.334751506 | 0.0001541 | 1 |
| 3717 | -0.483177937 | 0.0024763 | 1 |
| 51050 | -0.194717989 | 0.0010822 | 1 |
| 6857 | 0.191843246 | 0.0008986 | 1 |
| 8914 | 0.415690432 | 0.0020882 | 1 |
| 9435 | -0.334830405 | 0.003851 | 1 |
| 9466 | -0.352633849 | 0.0020738 | 1 |
| 10320 | -0.745477312 | 0.002974 | 0.9975 |
| 917 | -0.426583007 | 0.000965 | 0.9975 |
| 9266 | 0.519573495 | 0.003176 | 0.9975 |
| 10615 | 0.198293162 | 0.0050362 | 0.995 |
| 284403 | 0.380312382 | 0.004213 | 0.995 |
| 29068 | -0.368270993 | 0.0044634 | 0.995 |
| 3575 | -0.230389464 | 0.0044368 | 0.995 |
| 51542 | -0.435854751 | 0.0033308 | 0.995 |
| 9397 | -0.465282687 | 0.004968 | 0.995 |
| 124222 | 0.215444224 | 0.0053381 | 0.9925 |
| 2651 | -0.38202357 | 0.0020123 | 0.9925 |
| 355 | -0.415042057 | 0.0047985 | 0.9925 |
| 53335 | -0.214068049 | 0.0049202 | 0.9925 |
| 6689 | -0.285904865 | 0.0066772 | 0.9925 |
| 3009 | 0.285463706 | 0.0050661 | 0.99 |
| 4316 | -0.135238364 | 0.0056179 | 0.99 |
| 50512 | 0.518923557 | 0.0035305 | 0.99 |
| 79780 | -0.298220505 | 0.0067303 | 0.99 |
| 9768 | 0.300636688 | 0.0049514 | 0.99 |
| 1439 | -0.316298465 | 0.0045618 | 0.9875 |
| 26228 | -0.491064056 | 0.0070695 | 0.9875 |
| 27236 | 0.435404298 | 0.0055481 | 0.9875 |
| 4010 | 0.540592834 | 0.0061103 | 0.9875 |
| 4154 | -0.324960331 | 0.0071616 | 0.9875 |
| 5507 | 0.234489231 | 0.0056267 | 0.9875 |
| 57834 | 0.577157316 | 0.0031826 | 0.9875 |
| 79817 | -0.491311325 | 0.0056542 | 0.9875 |
| 9142 | 0.180858981 | 0.0111511 | 0.9875 |
| 3002 | -0.228111755 | 0.006624 | 0.985 |
| 3696 | -0.313392643 | 0.0057272 | 0.985 |
| 8065 | -0.533019252 | 0.0073025 | 0.985 |
| 9465 | -0.537983248 | 0.0072729 | 0.985 |
| 29121 | -0.645771382 | 0.008576 | 0.98 |
| 29909 | -0.409131519 | 0.007673 | 0.98 |
| 29969 | -0.353076659 | 0.0078326 | 0.98 |
| 55520 | -0.506547903 | 0.0070679 | 0.98 |
| 6573 | 0.310949442 | 0.0082659 | 0.98 |
| 6875 | -0.494176245 | 0.00569 | 0.98 |
| 8243 | 0.754816896 | 0.0085085 | 0.98 |
| 2568 | -0.10702058 | 0.0082194 | 0.9775 |
| 10744 | 0.464239824 | 0.0107382 | 0.975 |
| 23272 | -0.36245632 | 0.0090402 | 0.975 |
| 672 | 0.467232383 | 0.0073671 | 0.9725 |
| 10550 | -0.363486633 | 0.0109163 | 0.97 |
| 2841 | -0.326478621 | 0.0088192 | 0.97 |
| 3821 | -0.430649474 | 0.0073487 | 0.97 |
| 55095 | 0.414282966 | 0.0096675 | 0.97 |
| 9603 | -0.274737776 | 0.009657 | 0.97 |
| 9700 | 0.254075091 | 0.010268 | 0.97 |
| 2731 | -0.268986741 | 0.0118348 | 0.965 |
| 3512 | -0.10736056 | 0.0084197 | 0.965 |
| 54900 | -0.295157928 | 0.0159921 | 0.96 |
| 58526 | 0.301876167 | 0.0118793 | 0.96 |
| 894 | -0.27318081 | 0.0131634 | 0.96 |
| 54816 | -0.384990276 | 0.0134728 | 0.9575 |
| 6375 | -0.206229536 | 0.0133226 | 0.9575 |
| 833 | 0.42406671 | 0.0114703 | 0.9575 |
| 9537 | 0.383525477 | 0.0106673 | 0.9575 |
| 1021 | -0.480626627 | 0.0103222 | 0.955 |
| 146712 | 0.393733216 | 0.0137298 | 0.955 |
| 59338 | -0.269979745 | 0.0133504 | 0.955 |
| 10444 | 0.672419897 | 0.0120636 | 0.9525 |
| 10957 | -0.291573717 | 0.0141848 | 0.9525 |
| 116984 | -0.30928062 | 0.0127906 | 0.9525 |
| 152 | 0.5023562 | 0.0123241 | 0.9525 |
| 3820 | -0.247016532 | 0.0109157 | 0.9525 |
| 60468 | -0.576158784 | 0.013712 | 0.9525 |
| 6256 | 0.450199201 | 0.0120508 | 0.9525 |
| 64859 | -0.388489891 | 0.0117898 | 0.95 |
| 7466 | 0.25795395 | 0.0130998 | 0.95 |
| 1832 | -0.162250955 | 0.0142463 | 0.9475 |
| 23649 | 0.339528349 | 0.0163494 | 0.9475 |
| 27229 | 0.51634314 | 0.0141013 | 0.9475 |
| 55704 | -0.636168297 | 0.0130226 | 0.9475 |
| 64919 | -0.229117359 | 0.0137278 | 0.9475 |
| 10844 | 0.472115711 | 0.0128997 | 0.945 |
| 7178 | -0.352163711 | 0.0120623 | 0.945 |
| 9894 | 0.354325337 | 0.0151078 | 0.945 |
| 9986 | 0.324773459 | 0.0138022 | 0.945 |
| 9882 | -0.287049545 | 0.0152988 | 0.94 |
| 7067 | 0.551680545 | 0.0138777 | 0.9375 |
| 23549 | 0.351908607 | 0.0161551 | 0.935 |
| 23683 | -0.289317548 | 0.0169478 | 0.935 |
| 57579 | -0.683111416 | 0.0151795 | 0.935 |
| 10308 | -0.325176643 | 0.0180566 | 0.9325 |
| 22914 | -0.341179093 | 0.015293 | 0.9325 |
| 368 | 0.286212934 | 0.015103 | 0.9325 |
| 5243 | -0.284956599 | 0.018769 | 0.9325 |
| 5272 | -0.433524285 | 0.0154516 | 0.9325 |
| 7042 | -0.244945982 | 0.0155562 | 0.9325 |
| 23060 | 0.469400151 | 0.0149756 | 0.93 |
| 2322 | -0.275956449 | 0.0153028 | 0.93 |
| 3344 | -0.415916713 | 0.0155775 | 0.93 |
| 645 | 0.201076553 | 0.0148972 | 0.93 |
| 7561 | -0.43878933 | 0.0147879 | 0.93 |
| 83480 | -0.457392345 | 0.0147397 | 0.93 |
| 10643 | -0.571369872 | 0.0180568 | 0.9275 |
| 1880 | -0.20857608 | 0.0166367 | 0.9275 |
| 25842 | -0.308781941 | 0.0159503 | 0.9275 |
| 1773 | 0.69523989 | 0.0162666 | 0.925 |
| 4063 | -0.518180797 | 0.0160602 | 0.9225 |
| 5211 | 0.342625468 | 0.0177012 | 0.9225 |
| 55177 | 0.436699466 | 0.0162964 | 0.9225 |
| 6455 | 0.394697434 | 0.0164305 | 0.9225 |
| 9077 | -0.2433549 | 0.0181366 | 0.9225 |
| 26060 | -0.304549668 | 0.016776 | 0.92 |
| 56253 | -0.363499652 | 0.0180478 | 0.92 |
| 10578 | -0.341254639 | 0.0184944 | 0.9175 |
| 11170 | -0.449192419 | 0.0179954 | 0.9175 |
| 16 | 0.307706113 | 0.0145765 | 0.9175 |
| 2306 | 0.520746916 | 0.0188942 | 0.9175 |
| 29851 | -0.414355914 | 0.0178639 | 0.915 |
| 58513 | 0.333640623 | 0.0190011 | 0.915 |
| 6945 | 0.40961146 | 0.0162397 | 0.915 |
| 7368 | -0.275213665 | 0.0158445 | 0.915 |
| 80124 | -0.342233293 | 0.0190717 | 0.915 |
| 4982 | -0.22381963 | 0.0188617 | 0.9125 |
| 7110 | -0.302058761 | 0.019498 | 0.9125 |
| 22993 | 0.492127782 | 0.0192508 | 0.91 |
| 6018 | -0.404174485 | 0.0214473 | 0.91 |
| 10877 | 0.481068811 | 0.0192757 | 0.905 |
| 10892 | -0.429425967 | 0.0153653 | 0.905 |
| 1235 | -0.218300739 | 0.0225358 | 0.905 |
| 28964 | 0.292282055 | 0.0208919 | 0.905 |
| 65124 | -0.248807304 | 0.0192373 | 0.905 |
| 11046 | -0.376274933 | 0.020916 | 0.9025 |
| 6718 | 0.293189875 | 0.0168775 | 0.9025 |
| 1117 | -0.206967107 | 0.019512 | 0.9 |
| 221037 | -0.277184488 | 0.0204872 | 0.9 |
| 22873 | -0.370909008 | 0.0210179 | 0.9 |
| 3507 | -0.177183261 | 0.0215274 | 0.9 |
| 56938 | -0.368980221 | 0.0215148 | 0.9 |
| 57823 | -0.201163773 | 0.0195035 | 0.9 |
| 5998 | 0.538741552 | 0.0199311 | 0.9 |
| 79083 | 0.152680483 | 0.018546 | 0.9 |
| 3092 | 0.455010009 | 0.020906 | 0.8975 |
| 3383 | -0.320680998 | 0.022203 | 0.8975 |
| 5579 | -0.386132242 | 0.020871 | 0.8975 |
| 2664 | 0.279126081 | 0.0201697 | 0.895 |
| 3835 | 0.323152729 | 0.0174126 | 0.895 |
| 51188 | -0.32034974 | 0.0219961 | 0.895 |
| 51199 | -0.312992126 | 0.0213563 | 0.895 |
| 55231 | 0.271955961 | 0.0198708 | 0.895 |
| 931 | -0.247397237 | 0.0215433 | 0.895 |
| 5255 | 0.341968907 | 0.0189558 | 0.8925 |
| 593 | 0.513170224 | 0.0212572 | 0.8925 |
| 9675 | 0.311160853 | 0.0204554 | 0.8925 |
| 2296 | -0.159164155 | 0.0205966 | 0.89 |
| 5725 | 0.514646397 | 0.0219159 | 0.89 |
| 6814 | -0.266584049 | 0.0242739 | 0.89 |
| 79805 | -0.334990392 | 0.0207353 | 0.89 |
| 9555 | 0.383911887 | 0.0218357 | 0.89 |
| 26034 | -0.31644654 | 0.0231777 | 0.8875 |
| 27334 | -0.408056432 | 0.0203519 | 0.8875 |
| 79934 | 0.469065425 | 0.0214132 | 0.885 |
| 54149 | -0.431790164 | 0.0193112 | 0.8825 |
| 55196 | -0.215721108 | 0.0227661 | 0.8825 |
| 55862 | -0.194377846 | 0.0233795 | 0.8825 |
| 7030 | 0.637796523 | 0.0247257 | 0.8825 |
| 9669 | -0.417708093 | 0.0225701 | 0.8825 |
| 2018 | 0.40643606 | 0.0236945 | 0.88 |
| 5914 | 0.167488106 | 0.0232456 | 0.88 |
| 92249 | 0.479598218 | 0.0230219 | 0.88 |
| 6314 | -0.414233237 | 0.0233286 | 0.8775 |
| 9235 | -0.215258313 | 0.0252822 | 0.8775 |
| 9368 | 0.151566012 | 0.0221275 | 0.8775 |
| 23247 | 0.373041312 | 0.0245294 | 0.875 |
| 29127 | 0.221454584 | 0.0233018 | 0.875 |
| 3932 | -0.244107784 | 0.0246353 | 0.875 |
| 54606 | 0.431658923 | 0.0237203 | 0.875 |
| 55507 | 0.412369762 | 0.0253131 | 0.875 |
| 3315 | 0.146583092 | 0.0222413 | 0.8725 |
| 4929 | 0.154492955 | 0.0222182 | 0.8725 |
| 51514 | 0.20481441 | 0.0243982 | 0.8725 |
| 55909 | -0.348142139 | 0.0219433 | 0.8725 |
| 5829 | 0.341966852 | 0.0211544 | 0.8725 |
| 11064 | -0.303406578 | 0.0238799 | 0.87 |
| 157697 | -0.460973573 | 0.0256197 | 0.87 |
| 3702 | -0.175296727 | 0.023281 | 0.87 |
| 51316 | -0.175668972 | 0.0205026 | 0.87 |
| 9934 | -0.299420011 | 0.021563 | 0.87 |
| 3003 | -0.150208492 | 0.0231869 | 0.8675 |
| 5788 | -0.211777354 | 0.024388 | 0.8675 |
| 597 | -0.198098217 | 0.0267579 | 0.8675 |
| 29988 | 0.34575791 | 0.0266079 | 0.865 |
| 4942 | -0.196632255 | 0.0247937 | 0.865 |
| 7468 | 0.349418706 | 0.0234292 | 0.865 |
| 9841 | -0.363728778 | 0.0265235 | 0.865 |
| 11138 | -0.257769431 | 0.0269819 | 0.8625 |
| 3001 | -0.212479624 | 0.024064 | 0.8625 |
| 7019 | -0.34877263 | 0.0239925 | 0.8625 |
| 6197 | -0.330727114 | 0.0253014 | 0.86 |
| 146542 | 0.436311463 | 0.0268922 | 0.8575 |
| 3980 | 0.463625867 | 0.0264668 | 0.8575 |
| 55843 | -0.236228251 | 0.0232499 | 0.8575 |
| 29021 | 0.546021303 | 0.027875 | 0.855 |
| 80179 | 0.248032935 | 0.027204 | 0.855 |
| 151 | 0.356250502 | 0.0271094 | 0.8525 |
| 22888 | 0.572727134 | 0.0264032 | 0.8525 |
| 3394 | -0.257502456 | 0.0271686 | 0.8525 |
| 3705 | 0.260063621 | 0.0277535 | 0.8525 |
| 23194 | 0.236745501 | 0.02875 | 0.85 |
| 3570 | -0.367607408 | 0.0272347 | 0.85 |
| 80143 | -0.442067236 | 0.0293764 | 0.85 |
| 9409 | 0.480551893 | 0.0245266 | 0.85 |
| 1325 | 0.506576094 | 0.0265454 | 0.8475 |
| 2526 | -0.35142214 | 0.0268188 | 0.8475 |
| 27324 | 0.127332756 | 0.0267035 | 0.8475 |
| 57513 | 0.30892404 | 0.0274424 | 0.8475 |
| 2342 | 0.569425028 | 0.0274009 | 0.8425 |
| 23433 | -0.375948782 | 0.0263801 | 0.8425 |
| 2744 | -0.409546377 | 0.0288778 | 0.8425 |
| 55900 | -0.270948979 | 0.0265685 | 0.8425 |
| 25816 | -0.245860312 | 0.0258526 | 0.84 |
| 1870 | 0.326901068 | 0.030905 | 0.8375 |
| 390 | -0.16125677 | 0.0310161 | 0.8375 |
| 54847 | 0.179092151 | 0.0274743 | 0.8375 |
| 79767 | 0.206628477 | 0.0293386 | 0.8375 |
| 25839 | 0.30542495 | 0.026893 | 0.835 |
| 54556 | -0.38868859 | 0.0313864 | 0.8325 |
| 6683 | -0.348367296 | 0.0267012 | 0.8325 |
| 1741 | 0.40195322 | 0.0275745 | 0.83 |
| 23386 | 0.412839004 | 0.0292434 | 0.83 |
| 27074 | -0.128301275 | 0.0300137 | 0.83 |
| 7422 | 0.152666296 | 0.0309845 | 0.83 |
| 23075 | -0.33857316 | 0.0265768 | 0.8275 |
| 7360 | -0.292209337 | 0.0304494 | 0.8275 |
| 54726 | -0.713715652 | 0.03096 | 0.825 |
| 55317 | 0.423304852 | 0.0270375 | 0.825 |
| 5971 | -0.255344023 | 0.0280528 | 0.825 |
| 112 | 0.342739165 | 0.0324129 | 0.8225 |
| 220972 | -0.225819791 | 0.0312497 | 0.8225 |
| 27120 | 0.32083333 | 0.0345178 | 0.8225 |
| 8323 | -0.143385323 | 0.0319586 | 0.8225 |
| 60625 | 0.566402866 | 0.0288949 | 0.82 |
| 81553 | -0.385042558 | 0.0290508 | 0.82 |
| 8997 | 0.673983093 | 0.0311689 | 0.82 |
| 10101 | 0.334363799 | 0.0306163 | 0.815 |
| 414 | 0.394688514 | 0.033994 | 0.815 |
| 53918 | 0.401080329 | 0.033542 | 0.815 |
| 5613 | -0.263360986 | 0.0308602 | 0.815 |
| 346 | 0.48810864 | 0.0336494 | 0.8125 |
| 3561 | -0.198351778 | 0.0309112 | 0.8125 |
| 3981 | -0.341292211 | 0.0300439 | 0.8125 |
| 4957 | 0.341972697 | 0.0373822 | 0.8125 |
| 54866 | 0.438494635 | 0.0350129 | 0.8125 |
| 10179 | -0.278433435 | 0.0295574 | 0.81 |
| 54793 | -0.266941209 | 0.0306346 | 0.8075 |
| 6904 | 0.284543147 | 0.0289394 | 0.8075 |
| 9416 | 0.435483974 | 0.0334936 | 0.8075 |
| 221061 | -0.173986315 | 0.0349322 | 0.805 |
| 1609 | 0.488811097 | 0.0334109 | 0.8025 |
| 5368 | -0.437600672 | 0.0335136 | 0.8025 |
| 6363 | -0.112812921 | 0.0329074 | 0.8025 |
| 81537 | -0.189050348 | 0.0322027 | 0.8025 |
| 4281 | -0.161462134 | 0.0342643 | 0.8 |

These are 267 genes selected by the bootstrapping method.The p-values and cox coefficient are the average values of the genes in the 400 runs and the stability of each gene is the ratios of the gene which are significant across all the 400 runs.

**Table S2.** The performance of DPBM on random sampling sets

| Data sets | Hazard ratio | HR 0.95 CI - | HR 0.95 CI + | p-value |
| --- | --- | --- | --- | --- |
| Training set | 3.31 | 1.84 | 5.95 | 2.49E-04 |
| Test data set | 3.15 | 1.39 | 7.22 | 0.0082 |
| Independent data set | 2.48 | 1.26 | 4.91 | 0.015 |

**Table S3.** All the dysregulated pathways in the metastasis process to other organs

| KEGG pathways | Enrichment p-value | Candidate genes | Cox cofficient | Cox p-value | Stability | |
| --- | --- | --- | --- | --- | --- | --- |
| GLYCOLYSISGLUCONEOGENESIS | 0.0022 | 219 | 0.45 | 0.00037 | 1 |  |
|  |  | 224 | -0.23 | 0.0030 | 1 |  |
|  |  | 230 | 0.15 | 0.011 | 0.9625 |  |
|  |  | 2023 | 0.34 | 9.45E-05 | 1 |  |
|  |  | 2203 | -0.16 | 0.0074 | 0.9825 |  |
|  |  | 2597 | 0.28 | 0.0033 | 0.995 |  |
|  |  | 3939 | 0.37 | 0.00067 | 1 |  |
|  |  | 3945 | 0.14 | 0.019 | 0.91 |  |
|  |  | 5160 | 0.33 | 0.017 | 0.9275 |  |
|  |  | 5211 | 0.28 | 0.012 | 0.965 |  |
|  |  | 5214 | 0.24 | 4.56E-05 | 1 |  |
|  |  | 5236 | 0.20 | 0.018 | 0.92 |  |
|  |  | 5315 | 0.33 | 0.0013 | 1 |  |
| PENTOSEPHOSPHATEPATHWAY | 0.0015 | 230 | 0.15 | 0.011 | 0.9625 |  |
|  |  | 2203 | -0.16 | 0.0074 | 0.9825 |  |
|  |  | 2539 | 0.20 | 0.012 | 0.9475 |  |
|  |  | 5211 | 0.28 | 0.012 | 0.965 |  |
|  |  | 5214 | 0.24 | 4.56E-05 | 1 |  |
|  |  | 5226 | 0.25 | 0.030 | 0.8125 |  |
|  |  | 5236 | 0.20 | 0.018 | 0.92 |  |
|  |  | 6120 | 0.50 | 0.0098 | 0.9675 |  |
| FRUCTOSEANDMANNOSEMETABOLISM | 0.024 | 230 | 0.15 | 0.011 | 0.9625 |  |
|  |  | 2203 | -0.16 | 0.0074 | 0.9825 |  |
|  |  | 2762 | 0.29 | 0.0049 | 0.99 |  |
|  |  | 5210 | 0.46 | 0.013 | 0.9375 |  |
|  |  | 5211 | 0.28 | 0.012 | 0.965 |  |
|  |  | 5214 | 0.24 | 4.56E-05 | 1 |  |
|  |  | 8898 | 0.47 | 0.010 | 0.9575 |  |
| GALACTOSEMETABOLISM | 0.021 | 2584 | 0.30 | 0.016 | 0.93 |  |
|  |  | 2585 | 0.54 | 0.0096 | 0.965 |  |
|  |  | 2717 | -0.14 | 0.033 | 0.805 |  |
|  |  | 5211 | 0.28 | 0.012 | 0.965 |  |
|  |  | 5214 | 0.24 | 4.56E-05 | 1 |  |
|  |  | 5236 | 0.20 | 0.018 | 0.92 |  |
| GLUTATHIONEMETABOLISM | 0.0096 | 2539 | 0.20 | 0.012 | 0.9475 |  |
|  |  | 2730 | 0.26 | 0.020 | 0.8825 |  |
|  |  | 2879 | -0.42 | 0.0012 | 1 |  |
|  |  | 2882 | 0.21 | 0.017 | 0.9275 |  |
|  |  | 2946 | -0.19 | 0.030 | 0.8275 |  |
|  |  | 2947 | -0.16 | 0.0036 | 1 |  |
|  |  | 2950 | 0.20 | 0.0093 | 0.965 |  |
|  |  | 3418 | 0.22 | 0.0012 | 1 |  |
|  |  | 5226 | 0.25 | 0.030 | 0.8125 |  |
|  |  | 6241 | 0.21 | 0.0050 | 0.9925 |  |
| CELLCYCLE | 0.027 | 890 | 0.50 | 0.027 | 0.825 |  |
|  |  | 891 | 0.18 | 0.014 | 0.945 |  |
|  |  | 898 | 0.23 | 0.015 | 0.92 |  |
|  |  | 994 | 0.19 | 0.0045 | 0.995 |  |
|  |  | 1017 | 0.49 | 0.031 | 0.82 |  |
|  |  | 1019 | 0.29 | 0.023 | 0.865 |  |
|  |  | 1111 | 0.26 | 0.014 | 0.94 |  |
|  |  | 1869 | 0.22 | 0.024 | 0.8925 |  |
|  |  | 2810 | 0.19 | 0.0046 | 0.995 |  |
|  |  | 4175 | 0.25 | 0.022 | 0.8625 |  |
|  |  | 4176 | 0.23 | 0.022 | 0.8975 |  |
|  |  | 7027 | 0.32 | 0.0061 | 0.975 |  |
|  |  | 8556 | -0.22 | 0.018 | 0.93 |  |
|  |  | 9133 | 0.23 | 0.0014 | 1 |  |
|  |  | 9232 | 0.21 | 0.032 | 0.8225 |  |
|  |  | 10744 | 0.38 | 0.030 | 0.83 |  |
|  |  | 23594 | 0.19 | 0.015 | 0.9475 |  |
|  |  | 29882 | -0.41 | 0.026 | 0.865 |  |
| OOCYTEMEIOSIS | 0.036 | 367 | -0.11 | 0.026 | 0.845 |  |
|  |  | 891 | 0.18 | 0.014 | 0.945 |  |
|  |  | 898 | 0.23 | 0.015 | 0.92 |  |
|  |  | 1017 | 0.49 | 0.031 | 0.82 |  |
|  |  | 3480 | -0.24 | 0.019 | 0.9025 |  |
|  |  | 3708 | -0.23 | 0.012 | 0.965 |  |
|  |  | 5241 | -0.24 | 4.58E-05 | 1 |  |
|  |  | 5594 | 0.31 | 0.031 | 0.8275 |  |
|  |  | 5604 | 0.36 | 0.012 | 0.9625 |  |
|  |  | 6790 | 0.21 | 0.0098 | 0.9825 |  |
|  |  | 8945 | -0.44 | 0.013 | 0.9575 |  |
|  |  | 9133 | 0.23 | 0.0014 | 1 |  |
|  |  | 9232 | 0.21 | 0.032 | 0.8225 |  |
|  |  | 10744 | 0.38 | 0.030 | 0.83 |  |
|  |  | 29882 | -0.41 | 0.026 | 0.865 |  |
|  |  | 51806 | 0.11 | 0.0021 | 0.9975 |  |
| P53SIGNALINGPATHWAY | 0.015 | 891 | 0.18 | 0.014 | 0.945 |  |
|  |  | 898 | 0.23 | 0.015 | 0.92 |  |
|  |  | 901 | -0.18 | 0.012 | 0.97 |  |
|  |  | 1017 | 0.49 | 0.031 | 0.82 |  |
|  |  | 1019 | 0.29 | 0.023 | 0.865 |  |
|  |  | 1111 | 0.26 | 0.014 | 0.94 |  |
|  |  | 2810 | 0.19 | 0.0046 | 0.995 |  |
|  |  | 3486 | 0.19 | 0.021 | 0.8975 |  |
|  |  | 6241 | 0.21 | 0.0050 | 0.9925 |  |
|  |  | 9133 | 0.23 | 0.0014 | 1 |  |
|  |  | 27244 | -0.25 | 0.023 | 0.885 |  |
|  |  | 56475 | 0.27 | 0.0021 | 0.995 |  |
| BLADDERCANCER | 0.0026 | 1019 | 0.29 | 0.023 | 0.865 |  |
|  |  | 1869 | 0.22 | 0.024 | 0.8925 |  |
|  |  | 1956 | 0.25 | 0.020 | 0.9025 |  |
|  |  | 3576 | 0.21 | 0.00033 | 1 |  |
|  |  | 4312 | 0.10 | 0.0034 | 0.99 |  |
|  |  | 4318 | 0.095 | 0.029 | 0.82 |  |
|  |  | 4893 | 0.24 | 0.025 | 0.8525 |  |
|  |  | 5594 | 0.31 | 0.031 | 0.8275 |  |
|  |  | 5604 | 0.36 | 0.012 | 0.9625 |  |
|  |  | 7422 | 0.21 | 0.0017 | 0.9975 |  |

## Supplementary figures


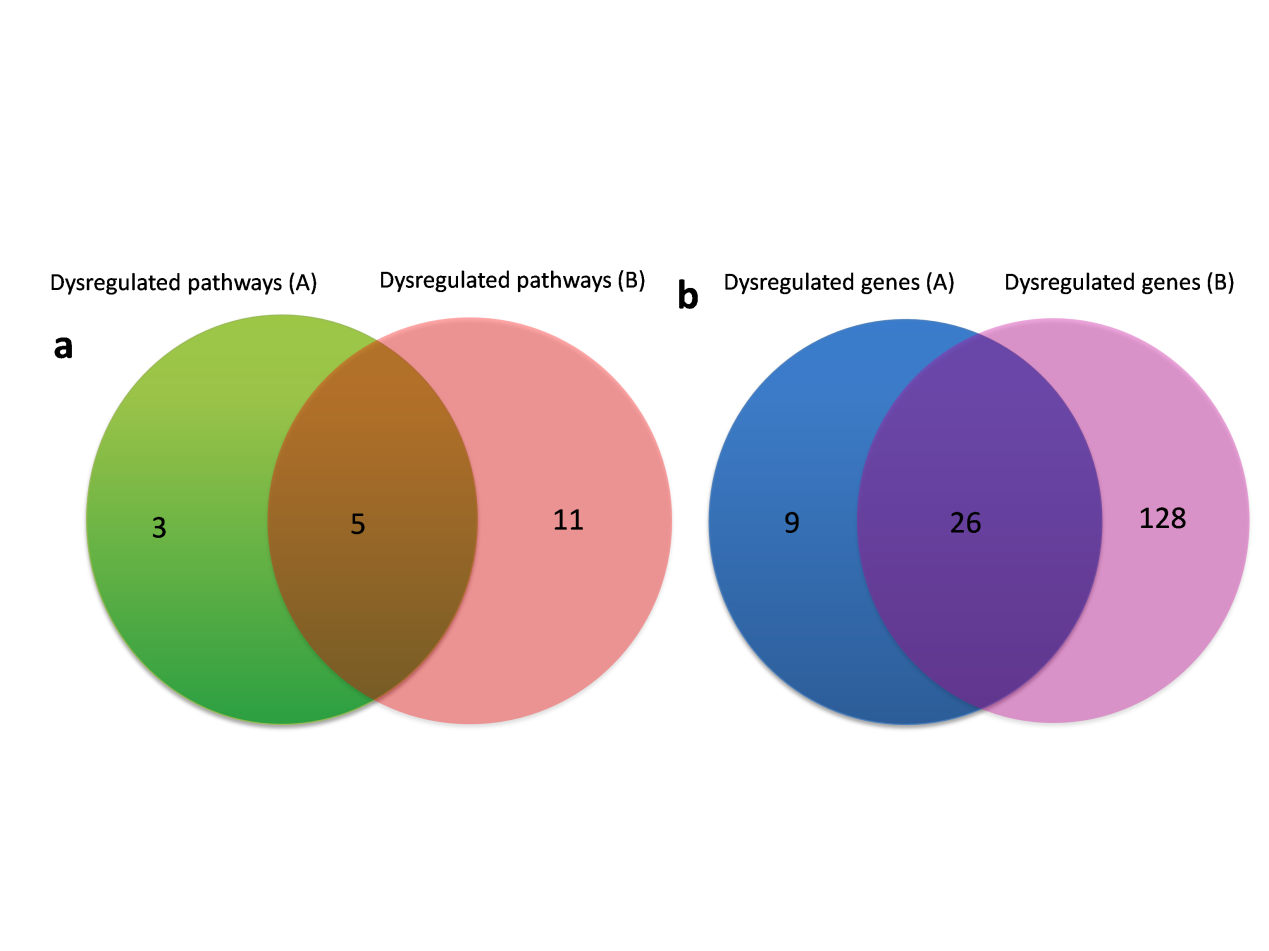


Figure S1. Overlap of dysregulated pathways (Figure S1.a) (p-value of 1.19E-07) and genes (Figure S1.b) (p-value less than 1.0E-17) based on different candidate selection method. In each sub-figure, elements in Set A are the dysregulated pathways / genes based on Cox proportional hazards regression, and elements in Set B are the dysregulated pathways /genes based on t-test.


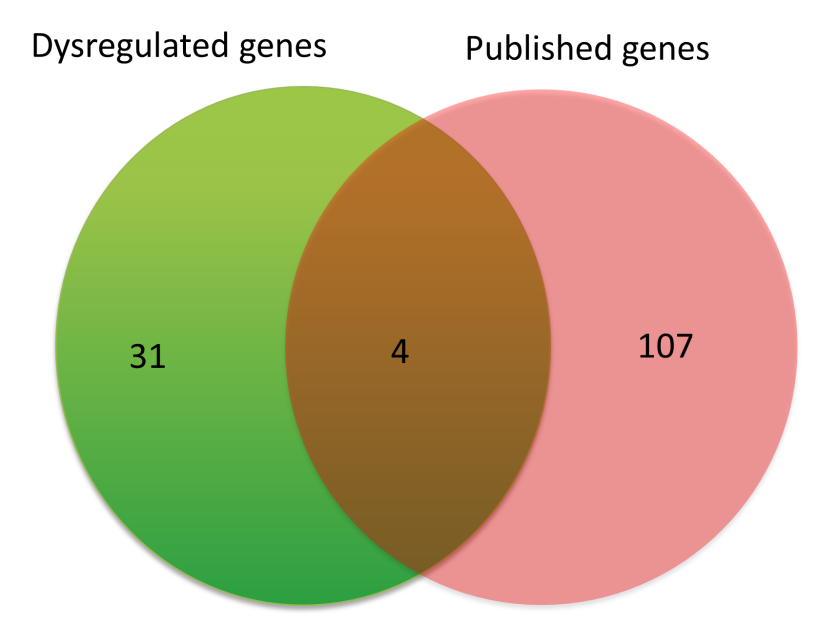


Figure S2. Intersection between the 35 dysregulated genes and the published bone-metastasis related genes (p-value = 3.98E-04).
